# Do Psychological Variables Affect Early Surgical Recovery?

**DOI:** 10.1371/journal.pone.0020306

**Published:** 2011-05-25

**Authors:** Michael N. Mavros, Stavros Athanasiou, Ioannis D. Gkegkes, Konstantinos A. Polyzos, George Peppas, Matthew E. Falagas

**Affiliations:** 1 Alfa Institute of Biomedical Sciences (AIBS), Athens, Greece; 2 Department of Obstetrics and Gynecology, Athens University School of Medicine, Athens, Greece; 3 Department of Surgery, Henry Dunant Hospital, Athens, Greece; 4 Department of Medicine, Henry Dunant Hospital, Athens, Greece; 5 Department of Medicine, Tufts University School of Medicine, Boston, Massachusetts, United States of America; The University of Queensland, Australia

## Abstract

**Background:**

Numerous studies have examined the effect of psychological variables on surgical recovery, but no definite conclusion has been reached yet. We sought to examine whether psychological factors influence early surgical recovery.

**Methods:**

We performed a systematic search in PubMed, Scopus and PsycINFO databases to identify studies examining the association of preoperative psychological variables or interventions with objectively measured, early surgical outcomes.

**Results:**

We identified 16 eligible studies, 15 of which reported a significant association between at least one psychological variable or intervention and an early postoperative outcome. However, most studies also reported psychological factors not influencing surgical recovery and there was significant heterogeneity across the studies. Overall, trait and state anxiety, state anger, active coping, subclinical depression, and intramarital hostility appeared to complicate recovery, while dispositional optimism, religiousness, anger control, low pain expectations, and external locus of control seemed to promote healing. Psychological interventions (guided relaxation, couple support visit, and psychiatric interview) also appeared to favor recovery. Psychological factors unrelated to surgical outcomes included loneliness, perceived social support, anger expression, and trait anger.

**Conclusion:**

Although the heterogeneity of the available evidence precludes any safe conclusions, psychological variables appear to be associated with early surgical recovery; this association could bear important implications for clinical practice. Large clinical trials and further analyses are needed to precisely evaluate the contribution of psychology in surgical recovery.

## Introduction

A good psychological state is an important index of health. Various psychological and psychosocial factors comprise what we call a “healthy psychology”, such as life satisfaction, optimism, self-esteem, and perception of social support. On the other hand, anxiety, stress, depression, and hostility reflect a less desirable psychological state which can affect one's health in various aspects [1]. So far, numerous studies have addressed the impact of psychological, psychosocial and personality factors on several health indices [2], [3]. Well-established evidence suggests that these factors may influence the short-term and long-term course of diseases, as well as patients' recovery and survival [4]–[6].

Scientists have been investigating the pathophysiological mechanisms through which psychology may influence the immunologic functions of human body, and several biobehavioral models have been proposed [7]. This field of medicine is called psychoneuroimmunology. More specifically, it has been stated that psychological variables could influence wound healing, thus surgical recovery, through direct and indirect paths. Directs paths include the effects of emotions on the stress hormones which regulate healing (cortisol, adrenaline, noradrenaline) [7]–[10], whereas indirect paths involve the psychology-influenced choice of anesthetic (choice of anesthetic may be influenced by the patient's psychological status) and general preoperative state of health (e.g. smoking, alcohol intake, obesity) [7].

Survival and long-term recovery after surgery can be influenced by numerous variables, apart from the preoperative psychological state; consequently its actual contribution is obscured when studying these outcomes. Thus, to examine the actual effects that psychological factors may have on surgical recovery, one should examine objectively measured, early postoperative outcomes. Despite the lack of evidence, one can assume that favorable early surgical recovery may be also associated with a shorter hospitalization and possibly long-term recovery. In this context, we sought to evaluate the existing evidence regarding the impact of psychological and psychosocial factors or interventions on the early postoperative outcomes of surgical patients.

## Methods

### Data sources

A systematic search of the literature regarding the association of psychological or psychosocial variables with the outcome of surgery was performed on PubMed, Scopus and PsycINFO databases (January 1970 to January 2010). The primary search was conducted with the following pattern: (psychological OR psychosocial OR personality) AND (surgery OR surgical OR postoperative*) AND (recovery OR outcome). Secondary searches used the following terms in various combinations: “psychological”, “psychosocial”, “personality”, “surgery”, “surgical”, “postoperative*”, “outcome*”, “recovery”, “prognosis”, “complication*”. We also sought to find potentially useful studies in the references of the relevant articles. We considered eligible all studies written in English, Spanish, German, French, Italian and Greek.

### Study selection

Three investigators (MNM, IDG, KAP) independently searched the literature and examined relevant studies for potential inclusion in this review. Any disagreement was resolved by consensus in meetings with all investigators. To be considered eligible, a study should examine the association of preoperative psychological or psychosocial status or intervention with an early objectively measured postoperative outcome in surgical patients. We considered eligible observational as well as interventional original studies, regardless of blinding and randomization. One exclusion criterion was set regarding the populations examined: having a diagnosed mental illness. Apart from that, studies in healthy volunteers and patients requiring elective or emergent surgery were eligible for inclusion. Unpublished studies reported as abstracts in conferences were not included in this review [11].

### Data extraction

We extracted data regarding the design (prospective or retrospective), the statistical analysis (univariate or multivariate), the population characteristics (number of participants, type of surgery, controls), the quality of the study (randomization, blinding), the psychological or psychosocial variables or interventions, the factors controlled in each analysis, the early postoperative outcomes measured and the related methodology, and the results of each study. When a study used both univariate and multivariate analyses for the same outcomes, the multivariate analysis data were extracted.

### Definition of psychological status and interventions

The psychological status was defined according to the definitions of each study. Most studies used the standardized psychometric scales to determine the patients' psychological status. No restriction was set in the psychological or psychosocial factors measured preoperatively. We included studies examining distress [12], depression [13], [14], loneliness [13], coping [12], [15], [16], anger expression and control [17], state and trait anger and hostility [14], [18], state and trait anxiety [15], [18], perceived stress [19], [20], perceived social support [14], optimism [14], [21], intramarital relationships [22], religiousness [14], expectations about recovery [15], health locus of control [15], worry about the operation [15], [16], [19], examinations-induced stress [23], and demented relative-induced stress [24]. Perceived stress and demented relative-induced stress were considered equivalent to trait anxiety, whereas worry about the operation and examinations-induced stress were considered equivalent to state anxiety. “State” and “trait” are defined as the sudden subversion and the baseline of one's emotional equilibrium respectively.

Similarly, no restriction was set regarding the psychological or psychiatric intervention in the studied groups. Examined interventions included guided relaxation [17], [25], [26], relationship-support or conflict visits [22], and psychiatric consults [27].

### Examined outcomes

To be considered eligible for our review, any study should examine the effect of psychological factors on at least one objectively measured, early postoperative surgical outcome. We excluded outcomes that were reported to be measured later than a month postoperatively. Also excluded were outcomes that were assessed by patients (such as perceived recovery or quality of life) and outcomes which could be directly or indirectly affected by the preoperative psychological status of the studied population regardless of the surgery (e.g. we excluded the analgesic requirements and the ambulation, since a depressed patient is expected to require more analgesics and have a delayed ambulation due to diminished drive). Moreover, studies reporting solely non-surgical outcomes (e.g. outcomes unrelated to the site of the operation) were not included. Eligible outcomes included wound inflammation or healing (assessed by peroxide stimulation test [23], [24], cytokine levels in wound fluid [19], or clinically by independent surgeons [13], [15], [17], [18], [22], [25]) and postoperative complications (wound infection, hematoma, postoperative fever etc) [12], [14], [16], [18], [20], [21], [26], [27]. Any of these outcomes is defined as an index of recovery.

Numerous studies examined the effect of psychological factors on the hospital or ICU length of stay. The length of stay is a useful index of physical recovery; however, it is multiply determined and influenced by individual behaviors, health care usage and associated costs [14]. On those grounds, the length of stay was not considered an eligible outcome.

## Results

**Figure 1:**
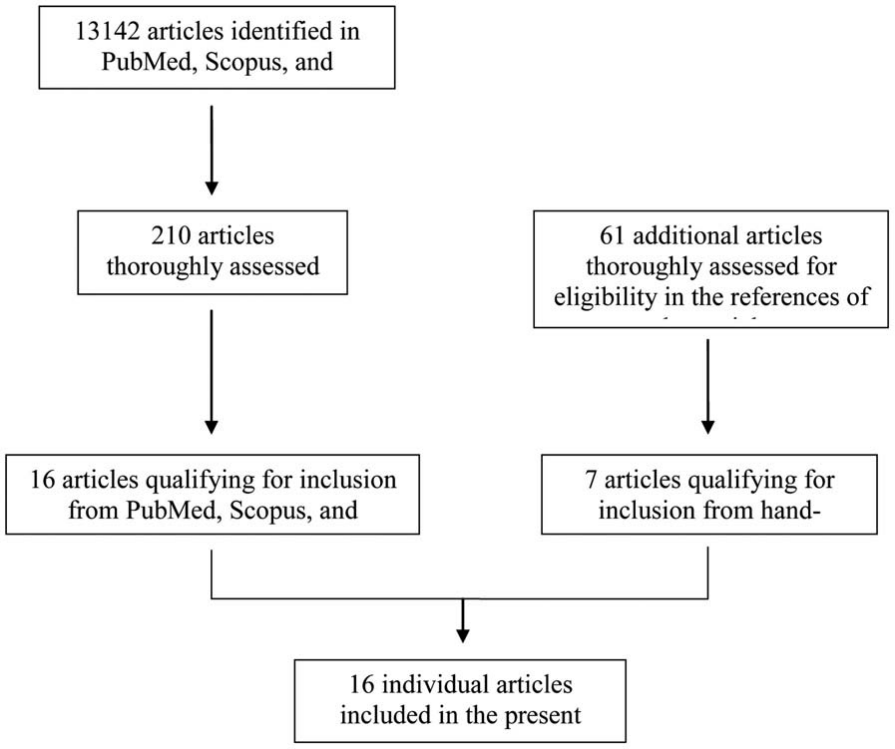
Flow diagram for reviewed studies.

A total of 16 studies were eligible for inclusion in the present review (Figure 1). Thirteen studies were observational (comparing two groups with different psychological status) and 5 were interventional (comparing the study group, which received the psychological intervention, with a control group); two of the studies had both an observational and an interventional design (in one study, the authors both assessed the volunteers' preoperative psychological status and applied an intervention [17]; in the other study they asked the volunteers to visit the clinic twice and performed two separate analyses [22]). In seven studies there was some degree of blinding, 4 of the interventional studies were randomized and 6 studies did not report any blinding or randomization. The median quality of the interventional studies was 2. Most of the studies were prospective in design (exception: [21]) and performed multivariate analyses (exceptions: [16], [23], [25], [27]).

The included studies enrolled a total of 1473 individuals (1071 patients and 402 volunteers, 1347 in observational and 266 in interventional studies [two studies had both an observational and an interventional design]). Of the 1071 patients, 861 received cardiac surgery [14], [18], [21], [26], [27], 173 general surgery [12], [16], [19], [20], [25] and 37 dental surgery [15]. Most of the procedures (1050 of 1071) were elective. Volunteers underwent standardized suction blister wounds [17], [22], punch biopsy wounds [23], [24], or oral mucosa wounds [13]. The examined outcome was assessed within the first postoperative month (8 of 16 studies did not report the time of assessment of outcomes but were included, since it was obvious that this was within the first postoperative month). Details on the measurement of each outcome are available in Table S1.

Fifteen out of 16 studies reported a significant association between at least one index of psychological status and surgical recovery (Table 1). The remarkable heterogeneity of the studies, along with the substantially different methodologies and definitions of outcomes did not allow for synthesis of the data with the methodology of meta-analysis. The only study showing no statistically significant association examined the effects of perceived life stress and cold pressor test on postoperative complications [20]. The scarce complications observed in this study did not allow for a direct effect of perceived stress to be shown, but even so, there was a significant association between reaction to cold pressor test and complications (p<0.05). Since reaction to cold pressor test is itself considered a measure of stress, this study also indirectly links stress with surgical outcomes.

**Table 1:** Impact of psychological variables on wound healing and postoperative surgical complications.

Psychological and psychosocial factors	Wound healing**		Complications**		No significant association
	Positiveimpact	Negative impact	Positive impact	Negative impact	
Trait anxiety^±^		[19] b, [24]			[15], [18] a, [19] b, [20] c
State anxiety^±^		[19] b, [23]		[18] a	[15], [16], [18] a, [19] b
Trait anger / hostility					[14], [18] a
State anger				[18] a	[18] a
Anger expression (in-out)					[17]
Anger control	[17]				
Coping (active - vigilant)				[12], [16]	[15]
Perceived social support					[14]
Optimism			[21] d		[14], [21] d
Distress (depression, anxiety, and stress)				[12]	
Depression		[12]			[14]
Loneliness					[12]
Intramarital hostility		[22]			
Religiousness			[14]		
Expectations about recovery (low pain expectations)	[15]				
Health locus of control (external)	[15]				
Relaxation intervention	[25] e		[26]		[17], [25] e
Support instead of conflict visit (couples)	[22]				
Psychiatric interview			[27]		

*In brackets are the references of the studies. An association was considered significant if p≤05. ^±^Demented relative-induced stress [24] and perceived life stress [19], [20] were considered equivalent to trait anxiety. Worry about the surgery [15], [16], [19] and exam-induced stress [23] were considered equivalent to state anxiety.

**Significant association of psychological factors with at least one aspect of wound healing or complications.

a State anger and anxiety were found to have no impact on clinical outcome (apart from complications) in this study.

b State anxiety and perceived stress were associated with some, but not all, markers of wound healing.

c Reaction to cold pressor test was significantly associated with postoperative complications.

d Only prolonged ventilation (out of 7 complications) proved to have significant association.

e Intervention was associated with less wound erythema, but not overall wound inflammation.

Including the above mentioned study, trait anxiety (or its equivalents, perceived stress and demented relative-induced stress) was examined in a total of 5 studies [15], [18], [19], [20], [24], showing a negative impact on recovery in 2 of them. Specifically, one study found perceived stress to result in attenuated healing (p<0.05) [19]. In addition, stress related to caring for demented patients in women was associated with a slower healing process (p<0.05) [24]. In contrast, no association was reported in the remaining two studies (on clinical assessment of the oral wound after dental surgery [15] and on postoperative complications after cardiac surgery [18].

State anxiety (or its equivalents, worry about the operation and examinations-induced stress) was examined in 5 studies [15], [16], [18], [19], [23]. One study reported an associated significant effect on postoperative complications after cardiac surgery (p<0.01) [18]. In two studies, increased state anxiety resulted in impaired wound healing after inguinal hernia repair (p<0.05) [19] and after a standardized wound in volunteers (p<0.001) [23]. However, the first study didn't find an association between psychological variables and clinical rating of outcome by two independent surgeons [18]. The remaining 2 studies­­ did not report any association between state anxiety and indices of recovery; however, recovery was associated with other psychological variables in those studies (described below) [15], [16].

Four studies examined the effects of anger or hostility [14], [17], [18], [22]. Anger expression (in versus out) was found to be unrelated to the outcomes, while anger control proved to result in faster wound healing (p<0.05)[17]. State anger had a significantly negative impact on complications (p<0.001), but no impact on clinical outcome [18]. Trait anger and hostility had no significant association with postoperative complications in two studies [14], [18], while intramarital hostility resulted in delayed wound healing (p<0.05) [22]. Decreased religious beliefs were found to increase complications (p<0.01) [14], while low pain expectations and external health locus of control resulted in decreased facial swelling (p<0.05) and faster healing (p<0.05), respectively, in patients undergoing 3rd molar extraction [15].

Coping (avoidance versus vigilance) was examined in 3 studies [12], [15], [16], 2 of which demonstrated increased complications in vigilant patients (p<0.05) [12], [16]. The third study found no association between coping and clinically assessed dental wound healing [15]. Subclinical depression, distress (defined as depression, anxiety and stress), and loneliness were studied in 3 articles [12]–[14]. Depression was found to have negative impact on healing (p<0.01) [13], but no significant impact on complications in another study [14]. Distress was associated with increased postoperative infections (p<0.05) [12], while loneliness had no effect on the speed of healing [13].

Perceived social support, assessed in one study, was not found to influence postoperative complications [14]. Two studies examined the effects of optimism, which were proved to be minimal, since in one study there was no significant association with complications [14] and in the other, optimism only reduced prolonged ventilation (p<0.05), of the 7 complications measured [21].

Psychological intervention of guided relaxation was assessed in three prospective randomized studies, which found it to have no [17] or minimal (only erythema was significantly increased, p<0.05) [25] effect on wound healing speed and wound inflammation, respectively, and a significant impact on the incidence of postoperative SVT (supraventricular tachycardia, p = 0.04), but not of severe SVT [26]. One study comparing the effect of a relationship-supporting versus a conflict-inducing psychiatric consult in couples observed slower healing after the conflict visit (p = 0.01) [22]. Another interventional study reported that one standard structured psychiatric interview before elective CABG (controls: no interview) in 33 patients resulted in less postoperative complications (p<0.05) [27].

## Discussion

Although the studies we reviewed were considerably heterogeneous, our findings suggest an association of preoperative psychological variables with early surgical recovery. Almost all studies found a psychological variable to have a statistically significant effect on one of the examined outcomes (Table 2). However, most studies also stated psychological factors that did not influence recovery from surgery significantly. Furthermore, psychological variables that were found to be significantly associated with recovery in some studies were found to be unrelated in other studies. This may be attributed to the limited number of participants in each study, which does not allow for all associations to be demonstrated.

**Table 2:** Overview of psychological factors and interventions examined in the reviewed studies.

**Factors associated with favourable recovery**	Anger control [17], low pain expectations [15], external locus of control [15], optimism* [21], religiousness [14], relaxation intervention* [25], [26], social support visit intervention [22], psychiatric interview [27]
**Factors associated with impaired recovery**	Trait anxiety* [19], [24], state anxiety* [18], [19], [23], depression* [12], intramarital hostility [22], state anger* [18], vigilant coping* [12], [16], distress [12]
**Factors not associated with recovery**	Trait anxiety* [15], [18]–[20], state anxiety* [15], [16], [18], [19], trait anger/hostility [14], [18], state anger* [18], anger expression [17], vigilant coping [15], perceived social support [14], optimism* [14], [21], depression* [14], loneliness [12], relaxation intervention* [17], [25]

In brackets are the references of the studies. An association was considered significant if p≤0.05.

*These factors were associated with some of the outcomes of the studies (but not all).

The effect of psychology on surgical recovery has been addressed in the past. Several studies have reviewed this association in various patient populations (cardiac surgery, dental surgery and others) and most of them acknowledge its influence [28]–[37]. A meta-analysis suggested that psychological preparation for surgery improves postoperative outcomes [28], while another review concurred that psychosocial factors are predictive of surgical outcomes [29]. Others have examined the cost-effectiveness of psychological interventions, which was confirmed through lower complication rate and decreased hospital and ICU length of stay [31]. However, our review is the first to examine solely objectively measured, early postoperative outcomes.

Apart from the outcomes examined in this study, numerous trials have investigated the impact of psychological factors and interventions on the hospital length of stay. Interventions such as randomizing surgical patients to a room with view of either a natural setting or a brick wall [38], or exposing the patients to therapeutic suggestions during general anaesthesia [39] proved to reduce hospital stay. However, the length of stay is a multiply determined measure, influenced by various factors irrelevant to our analysis; thus, such studies were not included.

This study should be interpreted in view of certain limitations. Initially, the vast variety of investigated psychological variables did not allow for a full search of the literature to be performed; however, we additionally searched the references of all relevant articles to minimize the chance of potentially missed studies. Moreover, the studies included suffer from heterogeneity in the populations, the factors controlled, the measuring of outcomes and the statistical analyses, a fact that prevents us from applying the findings of each to the general population. In particular, the differences between the studies' populations may account for part of the observed effects. For example, since volunteers undergoing standardized incisions or punch biopsy wounds are presumed to suffer less operation-related stress than patients undergoing major surgery, the effects of stress in the former group are considerably milder, and the association of psychological variables with surgical outcome is expected to be attenuated. In addition, the findings for many of the psychological variables are inconsistent across studies. Moreover, the psychometric tests used in some of the studies, despite their acknowledged credibility [40], [41], cannot substitute the gold standard of psychological evaluation, which is the clinical interview, and might pose risks if applied universally.

Our findings could bear important implications for clinical practice. The fact that groups exposed to psychological interventions had faster recovery or fewer complications, when compared to groups receiving standard care suggests that current standard care is far from optimal. Furthermore, since psychology has been shown to affect surgical outcomes, surgeons might consider arranging elective procedures in a time when the patient is in a better psychological state, or arrange for a psychological intervention to boost the patient's psychological status. In addition, in some cases, psychological variables might aid in the prediction of the surgical outcome.

In conclusion, although the significant heterogeneity of the available studies obscures the accuracy of our findings, an association between certain psychological variables and surgical recovery is suggested. Trait and state anxiety, state anger, active coping, subclinical depression, and intramarital hostility seem to complicate recovery, while dispositional optimism, religiousness, anger control, low pain expectations, and external locus of control can promote healing. Psychological interventions also appear to improve recovery. Loneliness and perceived social support, as well as anger expression, and trait anger didn't correlate significantly with the postoperative outcomes. Large randomized controlled trials and further analyses are needed to conclusively determine the potential contribution of psychological preparation of surgical patients to their postoperative recovery.

## Supporting Information

Table S1 Characteristics of the reviewed studies.
(DOC)
