# Supplementary material for: Do Psychological Variables Affect Early Surgical Recovery?
Source: PLoS One. 2011 May 25;6(5):e20306. doi: 10.1371/journal.pone.0020306 (PMC3102096; doi:10.1371/journal.pone.0020306)
Supplement: Table S1 — Characteristics of the reviewed studies. (DOC) [file pone.0020306.s001.doc]

**Table 1.** Characteristics of the reviewed studies.

| **First author, Reference** | **Characteristics of populations** | **Psychological/ psychosocial factors** | **Quality of study±** | **Factors controlled** | **Early outcomes** | **Results** |
| --- | --- | --- | --- | --- | --- | --- |
| Vollmer-Conna, 2009  [12] | Cholecystectomy, 29 pts (elective) | Distress, coping | B | *Analysis:* Age, smoking, alcohol, exercise, previous hospital admissions, general health issues  *Population is representative.* | Postoperative complications (fever, bile leak, wound hematoma, RTIs, UTIs etc) | More complications: Active coping (p<.05)  More infections: Distress (p<.05) |
| Gouin, 2008  [17] | Standardized vacuum blister wound, 98 volunteers | Relaxation training (57pts) vs control (41pts) | 2 [R] | *Analysis:* Age | Wound healing speed (daily monitoring for 8d) | No difference. |
|  |  | Anger expression | none | *Analysis:* Age, sex, BMI, health behaviors |  | Faster healing: anger NS, anger control (p=.013) |
| Bosch, 2007  [13] | Standardized oral mucosal wound on the hard palate, 183 volunteers | Depression, loneliness | B | *Analysis:* gender, age, ethnicity, smoking, alcohol, sleep, oral hygiene habits | Wound healing speed (serial photographing every other day) | Slower healing:  Depression (p=.007), loneliness (p=.13) |
| Kiecolt-Glaser, 2005  [22] | Standardized suction blister wound in volunteers, 42 couples | Intramarital relationships (high-hostility vs low-hostility) | none | *Analysis:* gender, visit/hostility  *No variance:* age, education, duration of marriage, positive or negative affect, health-related behaviors | Wound healing speed (daily monitoring) | Slower healing:  high-hostility couples (p=.03)  after conflict visit (p=.01) |
|  |  | Conflict visit vs support visit (same couples) | 1 |  |  |  |
| ­­­­­­­­­­­­­­­­Halpin, 2005  [21] | CABG, 565 elderly (elective) | Optimism (368 optimistic vs 197 pessimistic) | none | *Analysis:* previous CABG, renal failure, congestive heart failure disease, and cardiovascular disease  *No variance:* NYHA Class and preoperative risk factors of age, race, diabetes, and a family history of CAD. | Complications (reoperation for bleeding, local infections, prolonged ventilation etc) | Prolonged ventilation: pessimistic (p<.05)  All other complications (+ cumulative) : NS |
| Contrada, 2004  [14] | Cardiac surgery, 142 pts (121 elective) | Religiousness, depression, perceived social support, dispositional optimism, trait hostility & anger | B | *Analysis:* demographics (**age**, gender, marital status, and education), biomedical predictors (number of grafts, duration of anesthesia, surgical urgency, previous heart surgery, left-main stenosis, and comorbidity)  *Population is representative* | Complications (undefined) | More complications:  Less religious beliefs (p<.01)  Other 4 psychosocial factors: NS |
| Broadbent, 2003  [19] | Inguinal hernia patients, 37 pts (open, elective) | Perceived stress, worry about the surgery | none | *Analysis:* Age, gender, smoking, alcohol, sleep, exercise, anesthetic type | Wound healing (IL-1, IL-6, MMP-9 in wound fluid 20h post-op) | Lower IL-1: Increased perceived stress (p=.03), worry: NS  Lower MMP-9: Worry about the operation (p=.03), stress: NS  IL-6: all NS |
| Marucha, 1998  [23] | Standardized punch biopsy wound, 11 volunteers | Examination-induced stress  Wound in vacation vs 3d before exams | B | *No variance:* health-related behaviors | Wound healing (photograph, foaming after peroxide stimulation) | Slower healing: during exams (p<.001) |
| Stengrevics, 1996  [18] | Cardiac surgery, 94 pts (elective) | State and trait anxiety, state and trait anger | B | *Analysis:* Age, gender, priority, preoperative length of stay, NYHA classification, operation | Complications, clinical outcome (assessed by 2 surgeons) | More complications: State anger (p<.001), state anxiety (p<.01)  Clinical outcome: NS |
| Kiecolt-Glaser, 1995  [24] | Standardized punch biopsy wound, 26 volunteers | Demented relative-induced stress  13 study vs 13 control | B | *No variance:* health-related behaviors  *Analysis:* age, gender, income | Wound healing (photograph, foaming after peroxide stimulation) | Slower wound healing: caregivers (p<.05) |
| Leserman, 1989  [26] | Cardiac surgery, 27 pts (elective) | Relaxation training  13 study vs 14 control | 2 [R] | *No variance:* age, sex, education, employment, NYHA classification, time with disease, previous cardiac surgery, most psychological variables, type of surgery | Complications (ventricular arrhythmias) | Less SVT episodes: intervention group (p=.04)  Severe SVT episodes”: NS |
| Schindler, 1989  [27] | CABG, 33 pts (elective) | Psychiatric intervention  16 study vs 17 control | 1 [R] | *No variance:* Age, gender, marital status, job status, no of vessels bypassed, time on the bypass pump | Complications (undefined) | More complications: controls (p=.019) |
| Holden-Lund, 1988  [25] | Cholecystectomy, 24 pts (elective) | Relaxation with guided imagery  12 study vs 12 controls | 2 [R, B] | *No variance:* socioeconomic status, religion, racial distribution, hospital-representation | Wound inflammation (edema, erythema, exudation assessed by 2 surgeons) | Overall inflammation: NS  More erythema: controls (p<.01) |
| Linn, 1988  [20] | Inguinal hernia, 24pts (elective) | Perceived life stress, cold pressor test | B | *Analysis:* Age, sex, ethnicity, education, social class , marital status, self-assessed health, preoperative medications, details of the operation  *Population is representative* | Complications (hematoma, wound infections, wound erythema etc) | More complications: High-reactors in cold pressor test (p<.05), perceived life stress: NS |
| George, 1980  [15] | 3^rd^ molar extraction, 37 dental pts | Expectations about recovery, anxiety about recovery, trait anxiety, coping style, and health locus of control | none | *Analysis:* Age, sex, race, ethnic background, intraoperative sedation, surgeons’ differences | Facial swelling, healing (assessed clinically) | *Correlation analysis:*  Low swelling: low pain expectations (p<.05)  Faster healing: external locus of control (p<.05) |
| Cohen, 1973  [16] | General surgery, 59 pts (elective) | Coping (avoidance, vigilance), worry about surgery | none | *No variance:* Medical condition, surgical procedure, gender, surgeon, previous surgery | Minor complications (nausea, inability to void, headache, fever etc) | More minor complications: Vigilance (p<.05) |

All studies (except for [5]) were prospective. All studies (except for [8, 11, 12, 15]) used multivariate analysis. When a study used both univariate and multivariate analyses for the same outcomes, the multivariate analysis data were extracted.

± Quality assessment of observational studies referred to blinding of the investigators; interventional studies were assessed using a modified Jadad score.

**Abbreviations:** B= blinded, R= randomized, NS= non-significant, pts= patients, RTIs = respiratory tract infections, UTIs = urinary tract infections, vs = versus, BMI= body mass index, CABG= coronary artery bypass surgery, NYHA= New York Heart Association, CAD= coronary artery disease, SVT= supraventricular tachycardia.
